# Coexpressed Genes That Promote the Infiltration of M2 Macrophages in Melanoma Can Evaluate the Prognosis and Immunotherapy Outcome

**DOI:** 10.1155/2021/6664791

**Published:** 2021-03-08

**Authors:** Kexin Yan, Yutao Wang, Yuxiu Lu, Zhangyong Yan

**Affiliations:** ^1^Department of Dermatology, China Medical University, The First Hospital of China Medical University, Shenyang, Liaoning, China; ^2^Department of Urology, China Medical University, The First Hospital of China Medical University, Shenyang, Liaoning, China; ^3^Department of Pharmacy, Fuzhou No. 1 Hospital Affiliated with Fujian Medical University, Fuzhou, Fujian, China; ^4^Department of Stomatology, Fuzhou No. 1 Hospital Affiliated with Fujian Medical University, Fuzhou, Fujian, China

## Abstract

**Purpose:**

To improve immunotherapy efficacy for melanoma, a coexpression network and key genes of M2 macrophages in melanoma were explored. A prognostic risk assessment model was established for M2-related coexpressed genes, and the role of M2 macrophages in the immune microenvironment of melanoma was elucidated.

**Method:**

We obtained mRNA data from melanoma and peritumor tissue samples from The Cancer Genome Atlas-skin cutaneous melanoma (TCGA-SKCM). Then, we used CIBERSORT to calculate the proportion of M2 macrophage cells. A coexpression module most related to M2 macrophages in TCGA-SKCM was determined by analyzing the weighted gene coexpression network, and a coexpression network was established. After survival analysis, factors with significant results were incorporated into a Cox regression analysis to establish a model. The model's essential genes were analyzed using functional enrichment, GSEA, and subgroup and total carcinoma. Finally, external datasets GSE65904 and GSE78220 were used to verify the prognostic risk model.

**Results:**

The yellow-green module was the coexpression module most related to M2 macrophages in TCGA-SKCM; NOTCH3, DBN1, KDELC2, and STAB1 were identified as the essential genes that promoted the infiltration of M2 macrophages in melanoma. These genes are concentrated in antigen treatment and presentation, chemokine, cytokine, the T cell receptor pathway, and the IFN-*γ* pathway. These factors were analyzed for survival, and factors with significant results were included in a Cox regression analysis. According to the methods, a model related to M2-TAM coexpressed gene was established, and the formula was risk score = 0.25^∗^NOTCH3 + 0.008^∗^ DBN1 − 0.031^∗^KDELC2 − 0.032^∗^STAB1. The new model was used to perform subgroup evaluation and external queue validation. The results showed good prognostic ability.

**Conclusion:**

We proposed a Cox proportional hazards regression model associated with coexpression genes of melanoma M2 macrophages that may provide a measurement method for generating prognosis scores in patients with melanoma. Four genes coexpressed with M2 macrophages were associated with high levels of infiltration of M2 macrophages. Our findings may provide significant candidate biomarkers for the treatment and monitoring of melanoma.

## 1. Introduction

Melanoma is the most common type of skin tumor. Because access to early screening and primary health care varies globally, the incidence and mortality rates associated with melanoma vary widely [1]. In the past, advanced melanoma was scary because it spread quickly and became life-threatening. However, melanoma is one of the most immunogenic tumors and can respond vigorously to immunotherapy. Advanced melanoma thereby transformed into a new oncology model for solid cancer and could test immune checkpoint inhibitors.

Nevertheless, melanoma possesses various inhibitory mechanisms that often act synergistically to evade surveillance and attack by innate and adaptive immunity. For this reason, more effective treatments are needed to activate tumor-specific immunity [2]. Causes of melanoma, including escape from immune surveillance, continuous angiogenesis, and insensitivity to growth inhibitors, can be triggered by molecular or epigenetic mechanisms that activate oncogenes or tumor suppressor genes [3].

Macrophages can be stimulated by various microenvironments and can be polarized into different cell subtypes [4]. Traditionally, macrophages are classified into classically activated M1 macrophages and alternately activated M2 macrophages [5]. M1 macrophages can be induced by Th1 cytokines, lipopolysaccharide, and interferon *γ* (IFN-*γ*). These stimulate immune responses. Activated M1 macrophages phagocytose, destroy, and eliminate tumor cells, and then present antigens to T cells to induce adaptive immune responses [6]. M2 macrophages can be induced by IL-4, IL-10, IL-13, and TNF-*β*. They show immunosuppressive effects, characterized by reduced antigen presentation to T cells and cytokine production that stimulate Th2 responses. M2 macrophages also participate in promoting tumor growth [7].

Tumor-associated macrophages (TAMs) are among the main immune components of the tumor microenvironment (TME). Many clinicopathological studies have shown that TAMs are very similar to polarized M2 macrophages [8]. The recruitment of M1 macrophages during melanoma progression is far less than the increase of M2 macrophages. Moreover, M1 macrophages can be transformed into the M2 phenotype in the early stage of melanoma. As with other human cancers, M2 macrophage accumulation is a predictor of poor outcome. The distribution of M2 TAM in melanoma tissues is involved in avoiding tumor cell death and immune surveillance, inducing angiogenesis and tumor cell activity [9].

Weighted gene coexpression network analysis (WGCNA) is an analytical software package used for high-throughput microarrays or RNA-seq datasets. It constructs weighted gene coexpression networks, identifies gene modules, and identifies critical genes in the module [10]. Tumor-infiltrating immune cells are an essential part of the tumor microenvironment, related to tumor prognosis and response to treatment. CIBERSORT is a computational method for quantifying cell composition from a large number of gene expression profiles. CIBERSORT accurately estimates the immune components of tumor biopsies [11].

**Figure 1:**
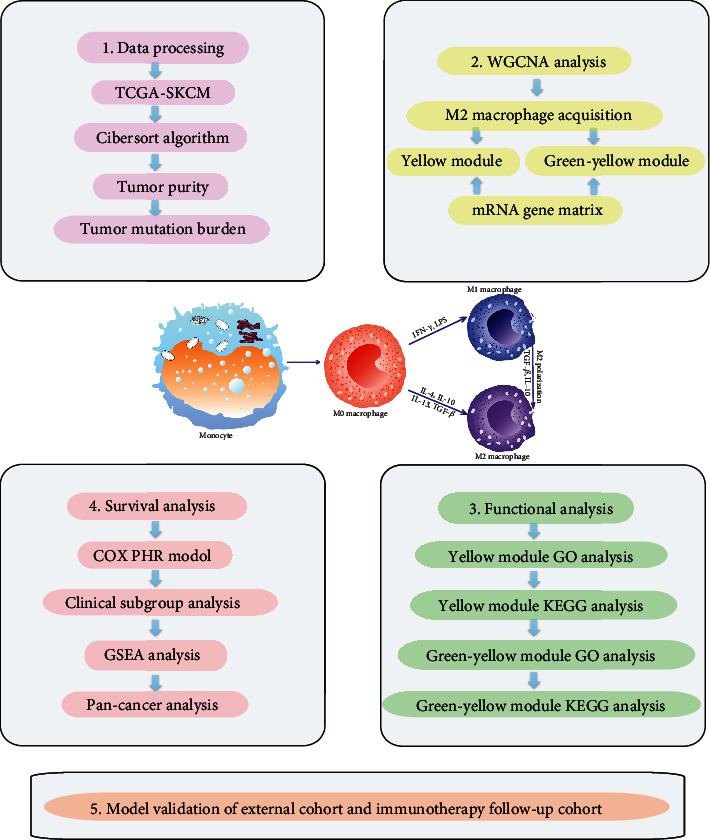
Flow chart to identify coexpressed genes that promote M2 macrophage infiltration in melanoma. (1) Data processing: 470 melanoma tissue samples were obtained from SKCM-FPKM. CIBERSORT algorithm was used to calculate M2 macrophage infiltration in melanoma tissue samples. Then, the tumor purity and mutation burden in the tumor immune microenvironment were analyzed. (2)WGCNA analysis: WGCNA was used to generate a coexpressed gene network to obtain coexpressed genes. Combined with the analysis of M2 macrophage content and mRNA gene matrix, the yellow module with the most negative correlation to M2 and the green-yellow module with the most positive correlation were selected. (3) Enrichment analysis: GO analysis and KEGG analysis were performed on the yellow module and the green-yellow module. (4) Survival analysis: the genes screened by WGCNA were analyzed for survival. According to these results, establish the Cox Proportional Regression model based on multiple factors and screen out the model's essential critical genes. TCGA-SKCM clinical subgroup analyses were performed on the prognostic model. GSEA analysis and pan-cancer analysis were performed on the essential genes. (5) Model validation: a prognostic scoring model related to the coexpression genes of M2 macrophages in melanoma was validated in the GSE69504 cohort and GSE78220 with immunotherapy follow-up cohort.

In this study, CIBERSORT and WGCNA were combined to preliminarily identify related modules and coexpressed genes of M2 macrophages in melanoma. A model was then established using multivariate Cox regression [12], and we performed survival analysis and subgroup evaluation of the model. Finally, the model's essential genes were analyzed using functional enrichment analysis and Gene Set Enrichment Analysis (GSEA) [13]. We also compared the immunohistochemical results of these genes in normal and tumor tissues. A flow chart is displayed in Figure 1, which illustrates the analytical logic of this article.

The new approach to immunotherapy for melanoma in the future will involve preventing the generation of M2, the transition from M1 to M2, and the reversal of TAM polarization, to reduce melanoma drug resistance and prevent the progression and recurrence. We hypothesized that this approach would affect the diagnosis and treatment of early and late metastatic melanoma.

## 2. Materials and Methods

### 2.1. Macrophage M2 and Immune Phenotype Calculation

We obtained TCGA-SKCM data from The Cancer Genome Atlas (http://cancergenome.nih.gov/), which contains 470 skin melanoma cancer tissue samples. GSE65904 [14] and GSE78220 [15] were also obtained from the GEO (http://www.ncbi.nlm.nih.gov/geo/) database whose platform is GPL10558 and GPL11154. We calculated macrophage M2 cell proportions based on the LM22 matrix using the CIBERSORT algorithm. Melanoma samples with *p* < 0.05 were considered to be significant and were taken into the subsequent analysis. The estimation of stromal and immune cells in malignant tumor tissues using expression data is a method that infers the fraction of stromal and immune cells using gene expression signatures [16]. We evaluated tumor purity in each melanoma sample base on this method. Tumor mutation burden per megabyte was also calculated [17].

### 2.2. Type M2 Macrophage Coexpression Network

A macrophage M2 coexpression network was generated using the weighted gene coexpression network analysis method. WGCNA is a system biology approach that converts coexpression correlations into connection weights or topology overlap values. We used this method to identify type M2 macrophage cell proportion coexpressing networks. We conducted a scale-free topology network, set the soft threshold at 5, *R* square = 0.81, slope = −1.83, and set the number of genes in the minimum module at 30. Each sample proportion of M2 macrophage was taken into the phenotype files in WGCNA analysis. In this manner, 19 M2 proportion coexpression networks were built. Sequentially, the genes with M2 macrophage correlation greater than 0.4 in the most relevant modules were identified.

### 2.3. Type M2 Macrophage-Related Module Function Enrichment

Genes in the M2 macrophage coexpression network were selected using ∣correlation coefficient | >0.4. We used Kyoto Encyclopedia of Genes and Genomes (KEGG) (https://www.genome.jp/kegg/) [18] and Gene Ontology (GO) (http://geneontology.org/) [19] to explore the biological functions of these genes.

### 2.4. A Risk Score Based on the M2 Macrophage Coexpression Network

We first carried out survival analysis on the genes with M2 correlations greater than 0.4 and then included the significant survival analysis factors into the multivariate Cox regression risk model. Next, we constructed a prognostic risk model based on the M2 macrophage content based on the coefficients. To evaluate the accuracy of our model, we divided the TCGA-SKCM cohort into different subgroups. We used survival stages to evaluate the prognostic value of the risk model in each subgroup. Also, we used the GSE65904 and GSE78220 queues to verify our conclusions.

### 2.5. Immune Phenotype Correlation

To explore the correlations between factors and immune phenotypes in the model, we included some immune indicators related to M2 macrophages. We performed Pearson tests on the factors in the score and tumor purity, immune score, tumor mutation burden, and CD8^+^ T lymphocytes.

### 2.6. The Human Protein Atlas (HPA) Database and GSEA Analysis

The HPA database was applied to determine the protein level differences for the genes in the risk model. GSEA analysis can interpret gene expression data and identify pathways associated with gene expression. The gene matrices of patients were divided into the high-expression group and the low-expression group according to the median ratio of M2 macrophages. Each analysis performed 1,000 genome substitutions. Gene sets with *p* < 0.05 and false discovery rate (FDR) < 0.05 were considered significantly enriched.

### 2.7. Timer

The coexpression factors of M2 type macrophages were clarified above; however, we only demonstrated their melanoma relevance. We speculated that this relationship might also be meaningful in other cancers; therefore, we used the TIMER database to explore these factors' correlations with M2 macrophages in other cancers [20].

## 3. Results

### 3.1. M2 Macrophage Content Acquisition

We obtained M2 macrophage proportions, tumor purity, stromal score, immune score, and tumor mutation burden from each melanoma carcinoma sample. Using the screening principle of *p* < 0.05, we obtained 214 melanoma samples accurately evaluated by M2 macrophages. By combining the microenvironment correlation score with the TCGA-SKCM mRNA expression files, we determined phenotype entry files of WGCNA. We also obtained melanoma samples in GSE65904 and GSE78220.

### 3.2. WGCNA Analysis

**Figure 2:**
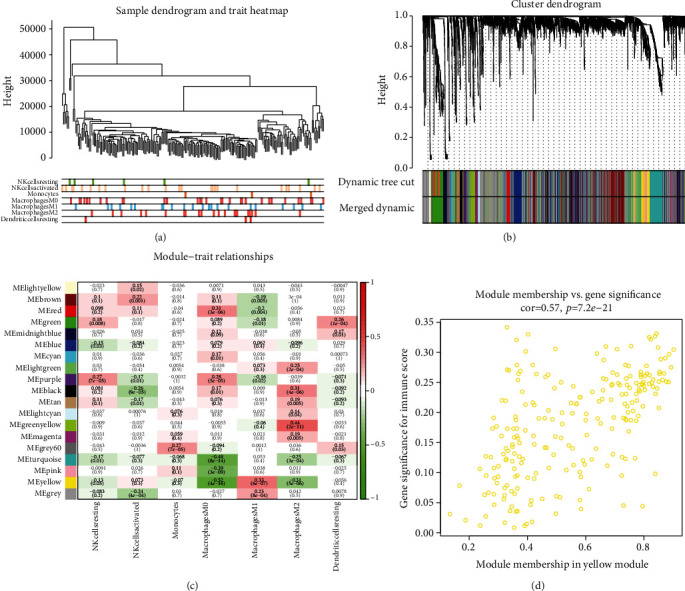
WGCNA analysis of SKCM-FPKM. (a) A hierarchical clustering tree is constructed by using the dynamic hybrid cutting method. (b) Each leaf represented one gene, each branch represented one coexpression module, and 19 coexpression modules were established. (c) The correlation coefficient between different phenotypes and coexpression modules was shown. The positive correlation between the green-yellow module and the M2 macrophage ratio was strongest (Cor = 0.44; *p* = 2*e*–11). The yellow module was correlated with the proportion of M2 macrophages (Cor = −0.31; *p* = 5*e*–06). (d) Heat map of correlation between factors in the green-yellow module (Cor = 0.64, *p* = 3.4*e*–13).

WGCNA analysis was performed on the TCGA melanoma cohort. We applied a dynamic hybrid cutting method to construct a hierarchical clustering tree (Figure 2(a)). Each leaf on the tree represents a gene, and each branch represents a coexpression module. A total of 19 coexpression modules were obtained (Figure 2(b)). Next, we calculated the correlation coefficients between each module and M2 macrophage proportions, and the yellow and green-yellow modules were determined according to the correlation coefficient (Figure 2(c)). The green-yellow module had the strongest positive correlation with the M2 macrophage proportion in TCGA melanoma cohort (Cor = 0.44; *p* = 2*e*–11). The magenta module had the highest negative correlation with the M2 macrophage proportion in the TCGA melanoma cohort (Cor = −0.31; *p* = 5*e*–06) (Figure 2(c)). Based on these findings, we supplemented the heat map of the correlation between the factors in the green-yellow module (Cor = 0.64, *p* = 3.4*e* − 13) (Figure 2(d)).

### 3.3. M2 Macrophage Coexpression Module Functional Analysis

**Figure 3:**
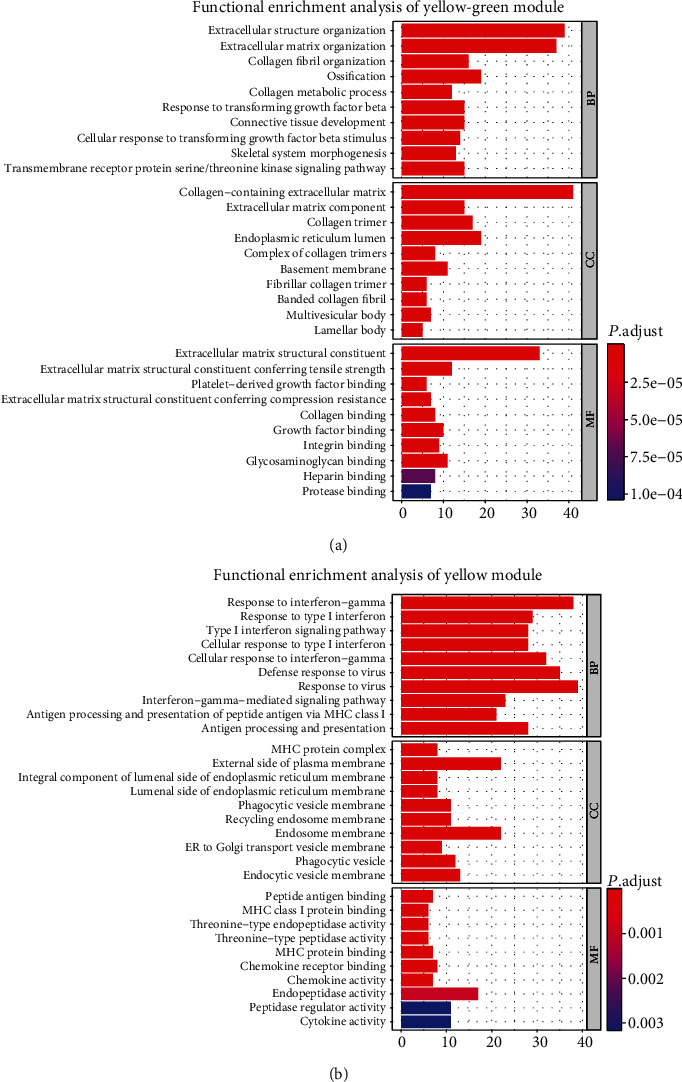
Functional enrichment analysis. (a) Functional enrichment analysis of the green-yellow module. In the biological process, essential genes are enriched in cells' response to transforming growth factor stimulation. (b) Functional enrichment analysis of the yellow module. In the biological process, the essential genes are concentrated in IFN-*γ*-mediated signaling pathway and antigen processing and presentation.

We determined the top 20 M2 macrophages positively coexpressing mRNA in the TCGA-SKCM green-yellow and yellow modules (Tables 1 and 2). The 20 M2 macrophage proportions positively coexpressing mRNA in the green-yellow module were most significantly enriched in response to transforming growth factor-beta. The 20 M2 macrophage proportion positively coexpressing the mRNA yellow module were most significantly enriched in response to IFN-*γ*, suggesting that these biological regulation functions might be positively related to M2 macrophage infiltrating the melanoma immune microenvironment (Figures 3(a) and 3(b)).

### 3.4. Clinical Outcome Analysis

**Figure 4:**
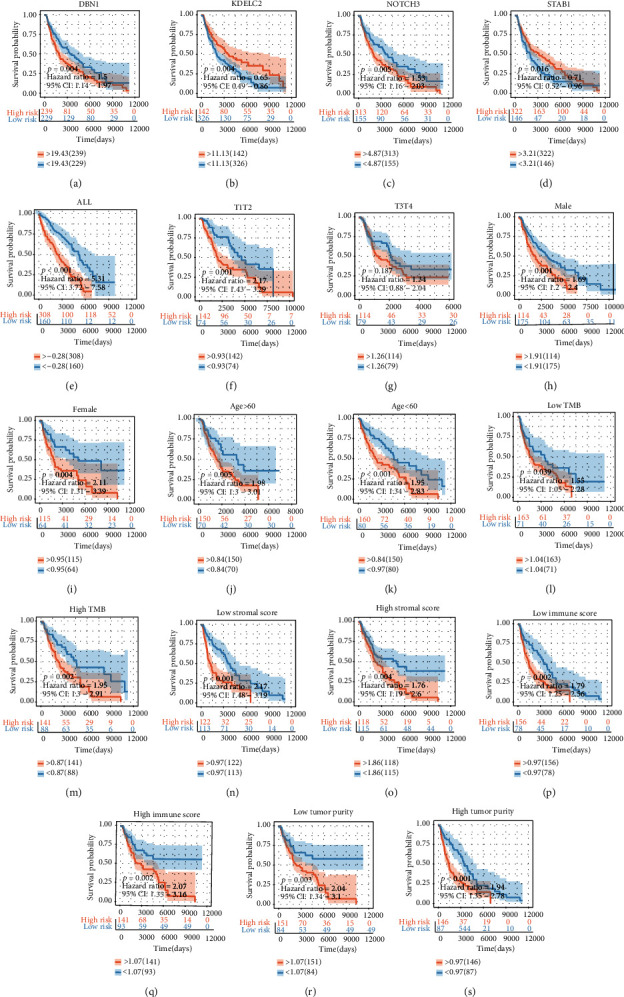
Survival analysis and subgroup analysis. (a–d) Independent prognostic analysis of four factors. (e–s) A subgroup assessment of the prognostic risk score model for M2 macrophage-associated coexpression factors.

To determine the overall survival outcome of these M2 macrophage coexpression genes, survival analysis was applied to determine their prognosis. The patients in the low-expression groups for DBN1 (TCGA: *p* = 0.004), KDELC2 (TCGA: *p* = 0.004), NOTCH3 (TCGA: *p* = 0.005), and STAB1 (TCGA: *p* = 0.016) showed survival risk compared to the high-expression groups (Figures 4(a)–4(d)). These results suggest that the coexpression genes in the green-yellow module act in protective roles against melanoma.

### 3.5. M2 Macrophage-Related Gene Risk Model and Subgroup Evaluation

An M2 macrophage coexpression gene Cox regression hazard proportion model was generated based on these melanoma prognosis protective factors.

(1) $$\begin{array}{c} \text{Risk}=0.25*\text{NOTCH}3+0.008*\text{DBN}1-0.031*\text{KDELC}2-0.032*\text{STAB}1. \end{array}$$

The risk score was evaluated in various subgroups, including age, gender, stage, tumor purity, and tumor mutation burden. The results were significant in these subgroups (Figure 4(e)–4(s)).

### 3.6. GSE65904 Verification

**Figure 5:**
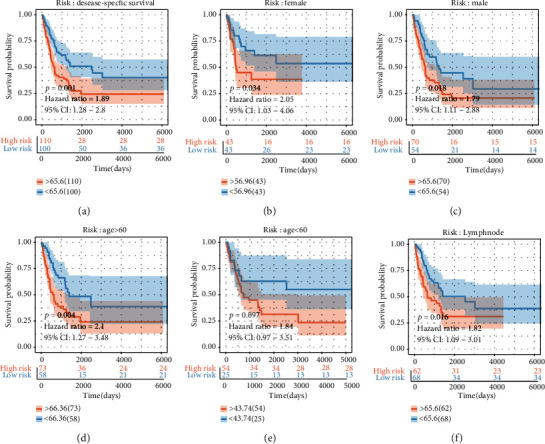
(a) Validation test and (b–f) subgroup evaluation of the GSE65904 cohort.

Considering the excellent test results, we verified the risk score in another queue. The M2 macrophage-related gene risk model was evaluated in GSE65904. The samples in the high melanoma risk group (GSE65904: *p* = 0.001, HR = 1.89) (Figure 5(a)) showed survival risk against the low-risk group, with the same results in the GSE65904 subgroups (Figures 5(b)–5(f)).

### 3.7. GSEA Analysis and HPA Analysis

**Figure 6:**
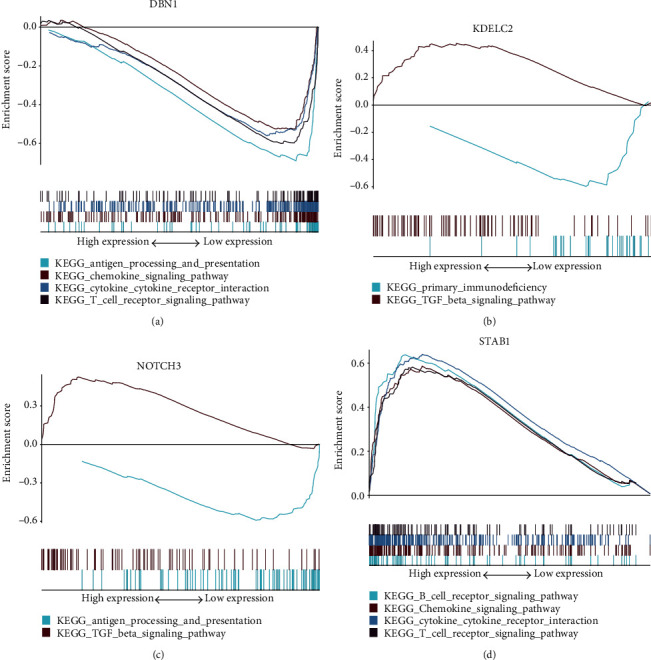
GSEA analysis. (a) DBN1 is enriched in antigen processing and presentation, chemokine signaling pathway, cytokine-cytokine receptor interaction, and T cell receptor signaling pathway. (b) KDELC2 is enriched in primary immunodeficiency, TGF-*β* signaling pathway. (c) NOTCH3 is enriched in the antigen processing and presentation, TGF-*β* signaling pathway. (d) STAB1 is enriched in the B cell receptor signaling pathway, chemokine signaling pathway cytokine-cytokine receptor interaction, and T cell receptor signaling pathway.

**Figure 7:**
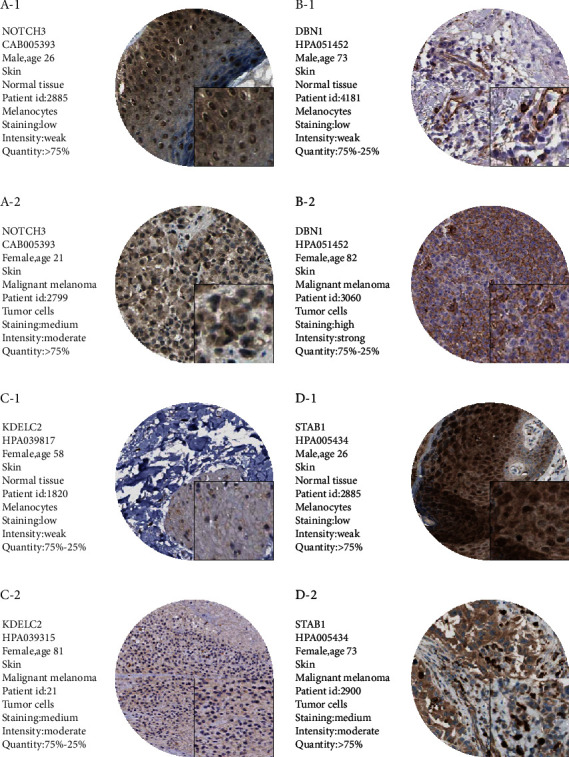
Differences in protein expression levels of NOTCH3, DBN1, KDELC2, and STAB1 were verified in the HPA database. In the immunohistochemical samples corresponding to each gene, the staining degree of melanoma tissue was higher than normal skin tissue.

Antigen processing and presentation, the chemokine signaling pathway, cytokine-cytokine-receptor-interaction pathway, and T cell receptor signaling pathway were related to the high-expression group in NOTCH3, DBN1, KDELC2, and STAB1 (Figure 6). Then, we found the immunohistochemical results of four independent factors in the risk scoring system in the HPA database, and we identified expression differences at the protein level (Figure 7).

### 3.8. Pan-cancer Analysis of M2 Macrophage Correlation

**Figure 8:**
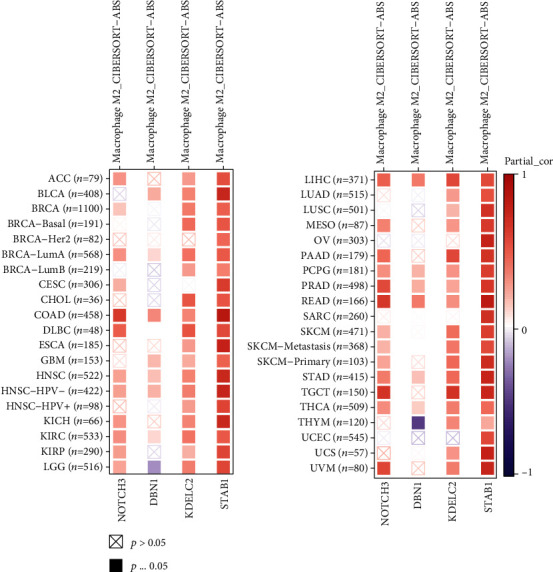
The correlation between NOTCH3, DBN1, KDELC2, and STAB1 and the M2 macrophage proportion in other cancer types was analyzed based on the TIMER database. In other types of cancer, the M2 macrophage proportion was also positively correlated in most cases, and STAB1 was most correlated with the M2 macrophage proportion in other types of cancer.

In these studies, we demonstrated the role of NOTCH3, DBN1, KDELC2, and STAB1 in melanoma patients. Next, we analyzed the correlations between these genes and M2 macrophage proportions in other types of cancers based on the TIMER database. The positive correlations to M2 macrophage proportion were determined in other types of cancers (Figure 8).

## 4. Discussion

M2-TAM plays a role in promoting tumor growth during the evolution of melanoma. Therefore, it is essential to study how to inhibit the expression of coexpressed genes of M2 macrophages in melanoma. The CIBERSORT package was used to analyze the proportion of the M2 component content in melanoma tissues. Combined with WGCNA, we found that the yellow-green coexpression module had the most positive correlation of M2 macrophage content. Subsequently, we performed functional enrichment analysis and GSEA analysis on the essential genes in the model. We found that they play essential roles in antigen treatment and presentation, chemotaxis, cytokine, the T cell receptor pathway, the IFN-*γ* pathway, and others.

In this coexpression network, COL1A2, COL5A1, ANTXR1, and other genes were positively correlated with M2 macrophages. Then, survival analysis was carried out for these factors, and factors with significant results were included in the subsequent Cox regression analysis. According to this method, a model related to the coexpression gene of M2-TAM was established.

The new model requires subgroup evaluation and external queue validation. Age, tumor mutation burden, immune score, tumor purity, and stroma score were grouped to analyze various prognoses. The results suggested that the prognostic risk assessment model of M2-related coexpressed genes had good prognostic evaluation ability in the TCGA-SKCM cohort. We also validated the prognostic risk score's ability to assess specific mortality in melanoma patients in the external datasets GSE65904 and GSE78220.

In summary, we identified four prognostic factors associated with M2 macrophages. We hypothesized that if these factors were closely related to M2, they might also be present in other cancers; therefore, we explored whether these factors also play roles in the coexpression of M2 in other cancers.

Macrophages are participants in the innate immune response and are the main components of immune cell infiltration in solid tumors. Macrophages were initially found to be involved in antitumor immunity; however, a growing body of evidence suggests that TAMs may also paradoxically enhance tumor development and metastasis [21]. These cells have the potential to possess both protumor and antitumor activities [22, 23]. During tumor progression, monocytes and macrophages are recruited to the tumor site to alter the tumor microenvironment. The escape of tumor cells from immune surveillance is a key to regulating tumor growth, survival, and metastasis. TAM, very similar to M2-polarized macrophages, is a crucial regulator of the tumor microenvironment. It has poor antigen presentation, inhibits the immune response of T cells by releasing immunosuppressive factors [24], and strongly induces the expression of programmed cell death 1 (PD-L1) [25]. TAM regulates the tumor microenvironment by secreting growth factors and proteolytic enzymes, allowing tumor cells to invade. During M1-M2 polarization, the tumor microenvironment is dominated by cytokines and growth factors. The release of immunosuppressive factors such as IL-10 and TGF-*β* can also polarize M1-M2 macrophages [26].

M2 type macrophages have been proved to play an important role in immunotherapy, and many emerging cases of combination of genetic engineering, nanomedicine, and immunotherapy have been reported [27, 28]. Lee et al. used a mixed peptide MEL-DKLA to induce the death of M2 macrophages, resulting in a slower tumor growth rate [29]. Xiao et al. developed a smart nanodrug that can trigger active targeting of M2-like macrophages only in acidic TME, repolarizing M2-like macrophages into M1 macrophages for cancer immunotherapy with low side effects [30]. Klichinsky et al. genetically engineered macrophages using chimeric antigen receptors. They found that the modified macrophages transformed M2 macrophages into M1 macrophages and expressed cytokine chemokines that upregulated the antigen presentation mechanism [31].

NOTCH receptor 3 (NOTCH3) acts as a signal receptor that controls cell fate. NOTCH3 synergistically acts with other NOTCH proteins to participate in the regulation of stem cells in various tissues and the plasticity of vascular smooth muscle phenotype in vascular remodeling. Depending on the type of tissue, transcription targets regulated by NOTCH may be oncogenic or tumor suppressor genes; however, NOTCH3 plays an oncogenic role. NOTCH3 signaling may play an essential role in tumor aggressiveness, maintenance, and chemotherapy resistance [32–34]. Some authors identified a relationship between NOTCH3 and macrophages. For example, NOTCH3 induced by nuclear factor kappa-B in injured renal epithelial cells maintains a proinflammatory environment, attracting activated macrophages to the site of injury [35]. During the culture of macrophages, it was found that they express various NOTCH pathway components, including all four receptors. NOTCH3 selectivity increased during macrophage differentiation [36].

NOTCH3 also plays an essential role in the development of melanoma. Pekkonen et al. conducted a series of experiments to identify specific proteins in melanoma cells that make cancer more aggressive when interacting with lymphatic cells. They found that lymphatic endothelial cells promote melanoma metastasis and invasion by relying on MMP14, NOTCH3, and *β*1 integrin [37]. The failure of melanoma treatment is due to tumor heterogeneity, especially subsets with stem cell-like characteristics. Hsu et al. found that NOTCH3 is a molecular switch that drives melanoma heterogeneity. The NOTCH3 signaling pathway may promote melanoma stem-like cells' plasticity and niche morphology in an environment-dependent manner [38]. Howard et al. reported that NOTCH3 is upregulated explicitly in melanoma, and the NOTCH3 signal transduction induced in melanoma cell lines can lead to enhanced tumor cell migration [39].

Although there is no clear evidence to elucidate the relationship between drebrin 1 (DBN1), KDEL (Lys-Asp-Glu-Leu) containing 2 (KDELC2) with M2 macrophages and melanoma, studies have shown that DBN1 and KDELC2 play essential roles in other cancers. The low expression of the DBN1 gene may be related to colon cancer cells' resistance to vincristine [40]. It is currently believed that DBN1 is involved in actin cytoskeletal recombination and inhibits the crosslinking and binding of actin filaments. DBN1 may play an essential role in cancer metastasis because actin recombination is an essential tumor cell migration and invasion process. Some authors used immunohistochemical techniques to measure the overexpression of DBN1 in colorectal cancer tissue lymph nodes and liver metastases in matching tissue sections [41]. Also, DBN1 is an independent prognostic indicator for luminal breast cancer related to endocrine treatment response and prognosis [42]. Lyama et al. found that, in terms of disease-free survival rate, the prognosis of patients with lung adenocarcinoma with strong DBN1 expression was significantly worse than that of patients with weak DBN1 expression [43].

KDELC2 is thought to be associated with apoptotic pathways [44]. Tsai et al. evaluated the inhibitory properties of glioblastoma stem cells and angiogenesis after knockout KDELC2 gene. They found that the activation of the NOTCH pathway induced glioblastoma, and the inhibition of the KDELC2 downregulated NOTCH3 receptor inhibited GBM invasive behavior [45].

Stabilin-1 (STAB1) is a receptor for endocytic stabilator expressed on alternating activated macrophages. It is expressed explicitly by discontinuous sinusoidal endothelial cells in the liver, spleen, and lymph nodes and M2 or activated macrophages in human malignancies. Schonhaar et al. analyzed STAB1 expression in melanocytic lesions and found the STAB1-positive blood vessels in all analyzed non-Langerhans histiocytic hyperplasia and melanocytic lesions [46]. The expression of STAB1 was found on the TAM of melanoma, and it can promote the tumor in the mouse model of melanoma. In the study of STAB1 expression on TAM in breast cancer, some scholars found that STAB1-mediated silencing of extracellular tumor growth suppressor was the mechanism of STAB1-induced tumor growth [47].

**Figure 9:**
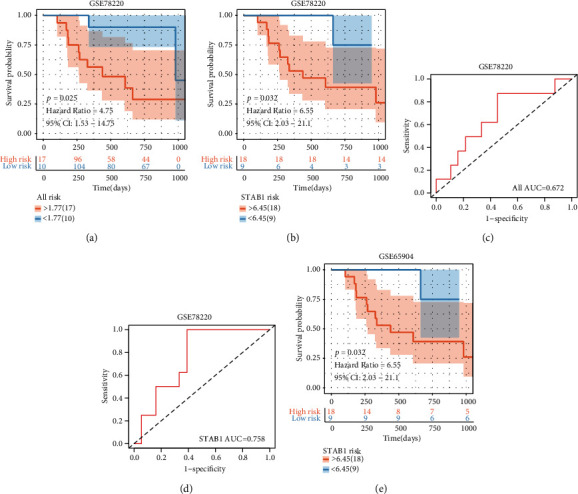
(a–d) External immunotherapy follow-up cohort validation of the model. In GSE78220, there was a significant difference in survival between the high-risk and low-risk groups, and patients with higher levels of STAB1 had a worse prognosis. (e) The low STAB1 expression group had a better effect on immunotherapy in GSE65904.

We know that CD8^+^T cell content can be used as one indicator to evaluate patients after immunotherapy and patients with more CD8^+^T cells can benefit more significantly from immunotherapy. The content of M2 macrophages in the tumor microenvironment was negatively correlated with the content of CD8^+^T cells. STAB1 was considered in this paper to have a strong positive correlation with M2 macrophages in melanoma. We verified in GSE65904 that the low STAB1 expression group had a better effect on immunotherapy (Figure 9(e)). This means that samples with a lower M2 macrophage content would benefit more from immunotherapy. Meanwhile, in GSE78220, the role of STAB1 in immunotherapy outcome follow-up was consistent with the positive correlation between STAB1 and M2 macrophages that we previously believed (Figures 9(a)–9(d)).

**Figure 10:**
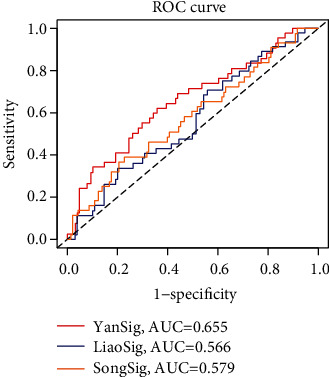
Comparison of models. The model in this article is compared with the model proposed by Liao et al. and the model proposed by Song et al. The model proposed in this paper has the highest AUC value and the best predictive ability.

We compared the prognosis model of M2 macrophages of melanoma in this paper with the prognosis model of immune-related proposed by other scholars [48, 49]. The area under curve (AUC) value of Yansig was 0.655, that of Liaosig was 0.566, and that of Songsig was 0.579 (Figure 10). Therefore, it can be seen that the prediction ability of the model in this paper is better. However, there were some limitations in this study. We selected a total of two queues from two databases for analysis, and more samples were needed to verify the scientific accuracy of the results. Although comprehensive bioinformatics analysis and layer by layer data verification were carried out in this study, further verification is still needed using in vitro experiments. Due to our research methods' limitations, the mechanism of these factors was not studied in depth in the scoring model. Also, some genes have been less studied, and few studies can be found; nevertheless, our findings may provide the basis for future research.

For patients with melanoma, especially those in advanced stages, including metastasis, combined immunotherapy should be emphasized. Conventional excision and targeted therapy may have neglected the combined effects of the immune microenvironment of melanoma. In summary, we found that NOTCH3, DBN1, KDELC2, and STAB1 were closely related to M2 macrophage infiltration in melanoma tissues. M2-TAM constitutes a part of the tumor microenvironment in malignant tumors, and it can promote the occurrence and progression of tumors. We believe that suppression of infiltrating M2 macrophages in tumor tissues is a new direction for melanoma immunotherapy. The Cox proportional hazard regression model established based on the coexpression genes of melanoma M2 macrophages may impact the prognosis and treatment of melanoma.

## Figures and Tables

**Table 1:** The top 20 gene significance for M2 macrophage cell-related genes in the green-yellow module.

ID	GS.T.cells.M2 macrophage	*p* value
COL1A2	0.505033449	3.46*E* − 15
COL5A1	0.475129912	2.15*E* − 13
ANTXR1	0.464696848	8.29*E* − 13
COL5A2	0.450124777	5.06*E* − 12
COL3A1	0.441765163	1.38*E* − 11
POSTN	0.432492676	4.04*E* − 11
NREP	0.432407501	4.08*E* − 11
LRP1	0.431348963	4.61*E* − 11
PXDN	0.426245907	8.20*E* − 11
VCAN	0.400316274	1.33*E* − 09
CD248	0.398530783	1.60*E* − 09
COL1A1	0.394554125	2.40*E* − 09
ADAM12	0.38940663	4.01*E* − 09
OLFML2B	0.382029469	8.27*E* − 09
NOTCH3	0.37934344	1.07*E* − 08
TGFB3	0.378152978	1.20*E* − 08
COL4A2	0.376079724	1.46*E* − 08
COL4A1	0.375167293	1.60*E* − 08
ISLR	0.37129622	2.30*E* − 08
FBN1	0.371019005	2.36*E* − 08

GS: gene significance.

**Table 2:** The top 20 gene significance for M2 macrophage cell-related genes in the yellow module.

ID	GS.T.cells.M2 macrophage	*p* value
SIRPG	-0.331	7.75*E* − 07
CD2	-0.326408965	1.12*E* − 06
HLA-F	-0.324754936	1.27*E* − 06
PSMB8	-0.321355618	1.67*E* − 06
CD7	-0.309946411	4.01*E* − 06
HAPLN3	-0.309157691	4.25*E* − 06
PDCD1	-0.307610411	4.78*E* − 06
NFKBIE	-0.305359497	5.65*E* − 06
BATF	-0.304894644	5.84*E* − 06
IRF1	-0.304084891	6.20*E* − 06
RNF114	-0.301049264	7.75*E* − 06
FBXO6	-0.297658896	9.91*E* − 06
GCH1	-0.296345108	1.09*E* − 05
PSMB9	-0.289993511	1.71*E* − 05
CD8B	-0.2883301	1.92*E* − 05
NUB1	-0.286883552	2.12*E* − 05
ETV7	-0.278476058	3.75*E* − 05
PARP14	-0.277295951	4.06*E* − 05
BTN3A1	-0.277173902	4.09*E* − 05
APOL3	-0.274631807	4.85*E* − 05

GS: gene significance.

## Data Availability

The dataset of this article was downloaded from the open-source databases TCGA and GEO.
